# β-Caryophyllene/Hydroxypropyl-β-Cyclodextrin Inclusion Complex Improves Cognitive Deficits in Rats with Vascular Dementia through the Cannabinoid Receptor Type 2 -Mediated Pathway

**DOI:** 10.3389/fphar.2017.00002

**Published:** 2017-01-19

**Authors:** Jie Lou, Zhipeng Teng, Liangke Zhang, Jiadan Yang, Lianju Ma, Fang Wang, Xiaocui Tian, Ruidi An, Mei Yang, Qian Zhang, Lu Xu, Zhi Dong

**Affiliations:** ^1^Chongqing Key Laboratory of Biochemistry and Molecular Pharmacology, School of Pharmacy, Chongqing Medical UniversityChongqing, China; ^2^Department of Neurosurgery, Chongqing Traditional Chinese Medicine HospitalChongqing, China; ^3^Department of Pharmacy, The First Affiliated Hospital of Chongqing Medical UniversityChongqing, China; ^4^The Experimental Teaching Center, Chongqing Medical UniversityChongqing, China

**Keywords:** β-caryophyllene, CB2, hydroxypropyl-β-cyclodextrin, inclusion complex, vascular dementia

## Abstract

This work was conducted to prepare β-caryophyllene-hydroxypropyl-β-cyclodextrin inclusion complex (HPβCD/BCP) and investigate its effects and mechanisms on cognitive deficits in vascular dementia (VD) rats. First, HPβCD/BCP was prepared, optimized, characterized, and evaluated. HPβCD/BCP and AM630 were then administered to VD rats to upregulate and downregulate the cannabinoid receptor type 2 (CB2). Results showed that HPβCD/BCP can significantly increase the bioavailability of BCP. Through the Morris water maze test, HPβCD/BCP can attenuate learning and memory deficits in rats. Cerebral blood flow (CBF) monitoring results indicated that HPβCD/BCP can promote the recovery of CBF. Moreover, molecular biology experiments showed that HPβCD/BCP can increase the expression levels of CB2 in brain tissues, particularly the hippocampus and white matter tissues, as well as the expression levels of PI3K and Akt. Overall, the findings demonstrated the protective effects of HPβCD/BCP against cognitive deficits induced by chronic cerebral ischemia and suggested the potential of HPβCD/BCP in the therapy of vascular dementia in the future.

## Introduction

Chronic cerebral hypoperfusion cases are widely distributed in elderly people (Xi et al., 2014). Considerable studies showed that a reduction in cerebral blood flow (CBF) might affect learning and memory processes, resulting in the development, and progression of dementia, such as vascular dementia (VD) (Farkas et al., 2007; Liu and Zhang, 2012). VD is a chronic syndrome caused by ischemic cerebrovascular diseases (Enciu et al., 2011), and is believed to be the second most common type of dementia (Gunstad et al., 2005). VD is often characterized by a progressive cognitive and behavioral deterioration, which is induced by blood loss in brain areas like hippocampus and white matter (Kalaria et al., 2004). To date, no specific drug can cure VD. Thus, an increasing number of studies have focused on the development of effective drugs to treat VD.

β-caryophyllene (BCP) is a natural sesquiterpene compound found in the essential oil of plants and has been reported to be a cannabinoid receptor type 2 (CB2) selective agonist (Gertsch et al., 2008; Al Mansouri et al., 2014). BCP can effectively prevent Alzheimer's disease (AD) (Cheng et al., 2014). Some studies revealed that BCP exerts a prominent protective effect on cerebral neurons (Assis et al., 2014) and thus might be beneficial in the prevention and treatment of cerebral diseases (Guo et al., 2014). BCP can also generate neuroprotective effects in ischemic models (Choi et al., 2013). However, little is known about the role of BCP in VD. Therefore, we examined the effects and mechanisms of BCP on VD rats.

The bilateral common carotid artery clamping (two-vessel occlusion, 2VO) was used to achieve chronic cerebral ischemia (Farkas et al., 2007). The CBF changed substantially after 2VO, thus, the CBF values can be used to evaluate the degree of ischemia and investigate the drug effects. Morris water maze (MWM) is acknowledged as a validated test for the evaluation of the spatial learning and memory retention abilities of rats (Morris et al., 1982; D'Hooge and De Deyn, 2001).

In previous studies, BCP exhibited poor water solubility and volatile sensitivity to light, oxygen, humidity, and high temperatures (Sköld et al., 2006). These conditions decrease the bioavailability and limit the pharmacologic action of the drug. Thus, our present study was conducted to develop a delivery system for BCP to improve its solubility and bioavailability. In particular, hydroxypropyl-β-cyclodextrin (HPβCD) was used in this study. β-Cyclodextrins (β-CDs) are widely used as complexing agents to enhance the stability, aqueous solubility, dissolution rate, and bioavailability of drug molecules (Mennini et al., 2014; Aiassa et al., 2015). HPβCD, a derivative of β-CD, is proven to be a safe vehicle that increases drug solubility and bioavailability (Brewster and Loftsson, 2007; Garnero et al., 2010). Various injectable products containing HPβCD are approved by the FDA for commercial use, such as Mitomycin from MitoExtra and Itraconazole from Sporanox (Maragos et al., 2009).

In the present study, an injectable β-caryophyllene/hydroxypropyl-β-cyclodextrin inclusion complex (HPβCD/BCP) was prepared for VD treatment, and its contributions on the alleviation of cognitive impairment in VD rats were investigated. First, HPβCD/BCP was prepared, characterized and administered to rats with chronic cerebral ischemia. MWM test, CBF monitoring, brain histology, and biochemical analyses were then performed to evaluate the effects of BCP on rat brains after 2VO. The results may provide an innovative approach with potential clinical benefits of BCP for VD.

## Materials and methods

### Materials

BCP was purchased from Adamas Reagent Co., Ltd (Basel, Switzerland). AM630 was purchased from Cayman Chemical Company (Michigan, USA). Naphthaline (internal standard, purity ≥99.0%) was purchased from Xiya Reagent (Chengdu, China). HPβCD was purchased from Shandong Binzhou Zhiyuan Biotechnology Co., Ltd. (Shandong, China). Olive oil was purchased from Xiya Reagent (Chengdu, China).

### Preparation and characterization of HPβCD/BCP

#### Preparation of HPβCD/BCP and determination of inclusion efficiency

The HPβCD/BCP was prepared through solution-stirring and freeze-drying method (Xu et al., 2014). HPβCD was weighed and placed in a vial with 10 mL water and magnetic stirring bar and then placed in a constant temperature magnetic stirring apparatus. After stirring for 1 h, the BCP dissolved in ethanol was added into the saturated HPβCD solution. The mixture was stirred continuously and then cooled down to room temperature (25°C). The cooled mixture was then filtered with a millipore filter (0.45 μm). The filtrate was frozen at −20°C for 5 h and then at −80°C overnight. Afterward, the samples were freeze-dried in a vacuum freeze dryer (Beijing Boyikang Laboratory Instruments Co., Ltd, Beijing, China) for 24 h. The HPβCD/BCP was obtained thereafter.

The inclusion efficiency of HPβCD/BCP was determined by a gas chromatography (GC) (Liu et al., 2013). A gas chromatograph (GC2014C, SHIMADU, Japan) with a flame ionization detector and a Wonda Cap 5 (30 m × 0.25 nm × 0.25 μm) capillary column was used. The HPβCD/BCP lyophilized powder was dissolved in 1.001 mg/mL naphthaline-ethanol solution and then detected by the GC. The vaporizing chamber temperature was set at 250°C. The oven temperature was initially set at 100°C, programmed to 140°C at 15°C/min, held for 0.5 min, followed by 30°C/min to 270°C and then finally held for 5 min. Hydrogen was used as carrier gas at a flow rate of 1.62 mL/min. The sample volume was 1 μL, and the inlet had a split ratio of 20:1. The inclusion efficiency % was calculated as follows:

Inclusion efficiency (%) = (mass of BCP in HPβCD/BCP)/(mass of added BCP).

#### Optimization of the prescription

A central composite design (Lou et al., 2014) was used to elucidate the main effects and interactions of the parameters, such as material inputting ratio (X1), reaction temperature (X2), and reaction time (X3). The inclusion efficiency was assigned as the indicator for the selection of the optimum formulation. A three-factor and five-level factorial design was employed for the optimization procedure with different material inputting ratios, reaction temperature, and time as prime selected independent variables. The values of the five coded levels of the three factors were then assumed after preliminary trials. The values are presented in Table 1. The inclusion efficiency of each formulation was measured as response values. Design-Expert software (8.05b) was used to generate and evaluate of the statistical experimental design.

**Table 1:** **Experimental design of independent parameters in the Box-Behnken design**.

**Independent parameters**	**Symbol**	**Range and level**				
		**−1.682**	**−1**	**0**	**1**	**1.682**
Material inputting ratio (n/n)	X1	0.2	0.36	0.6	0.84	1
Reaction temperature (°C)	X2	30	38	50	62	70
Reaction time (h)	X3	1	1.8	3	4.2	5

#### Characterization of HPβCD/BCP inclusion complex

The HPβCD/BCP was characterized by ultraviolet spectrophotometry (UV), differential scanning calorimetry (DSC) and Fourier transform infrared spectroscopy (FT-IR). The formation of HPβCD/BCP was determined using a UV–vis spectrophotometer (UV-2600, SHIMADZU, Japan). HPβCD (21 mg) and BCP (10 μL) were dispersed separately in 10 mL distilled water to obtain the HPβCD and BCP solutions, respectively. HPβCD/BCP lyophilized powder (22 mg) was added to 10 mL distilled water to obtain the HPβCD/BCP solution. HPβCD (20 mg) and BCP (10 μL) were added into a mortar, and then ground homogeneously. The ground powder was dissolved in 10 mL distilled water to obtain a physical mixture solution. All the samples above were centrifugated to obtain their supernatants. The supernatants were scanned in the range of 200–800 nm to obtain the UV–vis absorption spectrum.

DSC analysis of HPβCD, BCP, their physical mixture and HPβCD/BCP were performed using STA 449C thermal analyzer (Netzsch Corporation, Germany). Samples were weighed accurately and sealed in aluminum pans. They were then placed in the instrument and heated at a rate of 15°C/min from 40 to 350°C under a constant flow (25 mL/min) of nitrogen gas. An empty sealed pan was used as a reference (Tang et al., 2015).

Subsequently, HPβCD, BCP, the physical mixture and HPβCD/BCP were characterized using iS50 FT-IR (Nicolet, Thermo Scientific, USA). The FT-IR spectrum of each sample was collected from 4000 to 400 cm^−1^. HPβCD, the physical mixture and HPβCD/BCP were ground and mixed with spectrograde KBr powder at a mass ratio of 1:100. The resulting mixture was pressed forcibly into round disks with 8 mm diameter. BCP was dropped and spread on a KBr disk uniformly. The FT-IR spectra of all the samples were analyzed using the OMNIC 9.2 spectrophotometer software.

#### *In vitro* dissolution study

*In vitro* dissolution studies of HPβCD/BCP were performed according to Pharmacopoeia of the People's Republic of China (2010 Edition, Part 2, Appendix XC. No.1 method; National Pharmacopoeia Committee, 2010) by using a ZRS-6G Dissolution Apparatus (Tiandatianfa Science and Technology Co., Ltd, Tianjin, China). Phosphate buffer solution (PBS, pH 6.8) was used as release medium. To demonstrate the dissolution of the inclusion complex, the BCP–HPβCD physical mixture were used as contrast. The HPβCD/BCP lyophilized powder and the physical mixture were added into the rotating baskets and installed onto the dissolution apparatus. Subsequently, the baskets were placed into the PBS and stirred at 50 rpm at 37 ± 0.5°C. Approximately 5 mL of the release medium was drawn at appropriate intervals (5, 10, 20, 30, 45, 60, 90, 120, 180, 300 min), and replaced with 5 ml fresh medium simultaneously. All samples were centrifuged at 12,000 rpm for 5 min and then submitted to UV analysis at 205 nm upon proper dilution. The release profile of the formulation was expressed as cumulative release percentage vs. time.

#### *In vivo* experiments protocol and pharmacokinetic analysis

*In vivo* experiments were performed on SD rats. Rats were provided by the Laboratory Animal Center, Chongqing Medical University, China. The rats were housed in cages with constant temperature (22°C) and humidity (55%) and under a 12 h light–12 h dark cycle (lights on 06.00–18.00 h). The rats had free access to food and water. The experiment protocol was approved by the Animal Experimental Committee, Chongqing Medical University. Rats were randomly divided into two groups with five rats each. The first group (HPβCD/BCP group) received HPβCD/BCP by intraperitoneal injection. The second group (BCP-olive group) received BCP-olive solution by intraperitoneal injection. Up to 0.5 mL blood samples were collected from the orbit venous plexus of each rat and transferred separately into heparinized eppendorf tubes at predetermined time points (0.167, 0.333, 0.5, 0.75, 1, 1.5, 2, 3, 4, 6, 8, 10, and 12 h) after drug administration.

Blood samples were centrifuged at 12,000 rpm for 5 min to separate the plasma. The plasma samples were stored in the refrigerator at −20°C. Before analysis, 100 μL plasma, 90 μL ethyl acetate, and 10 μL internal standard naphthaline (5.035 μg/mL) were added into a centrifuge tube and vortexed for 5 min. Each sample was then centrifuged at 3200 rpm for 10 min and the supernatant (60 μL) was collected for GC analysis. The pharmacokinetic parameters of BCP-olive and HPβCD/BCP in SD rats were calculated using a DAS2.0 practical pharmacokinetics program. A non-compartment model was used to analyze the *in vivo* distribution of the drug.

### Surgery

The VD rat model was established by 2VO for 4 weeks. The rats were weighed and then anesthetized with chloral hydrate (3.5 mL/kg, i.p.). After disinfection, a midline neck incision was performed and bilateral common carotid arteries of rats were exposed and ligated with 4-0 type surgical silk to induce cerebral ischemia. The sham-operated rats were operated using the same procedures except ligation. During the surgery rats were maintained at 37.0 ± 0.5°C.

### Drug administration and experimental protocol

**Figure 1:**
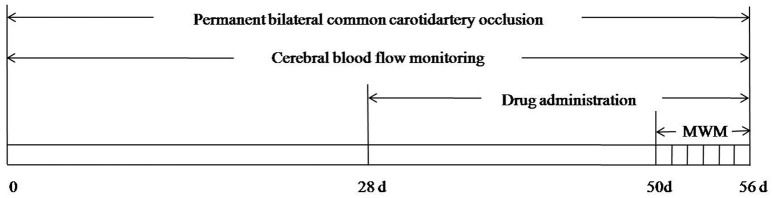
**Schematic demonstrates overall experimental process**.

A total of 84 adult male SD rats weighing 300–350 g were randomly divided into sham-operated group (sham group), 2VO group, HPβCD/BCP treated groups and AM630 treated group (3 mg/kg). The sham and 2VO groups were treated with normal saline containing HPβCD. The HPβCD/BCP treated groups were further subdivided into three groups, namely, high-, middle-, and low-dose groups treated with 144, 48, and 16 mg/kg BCP, respectively. AM630 was dissolved in dimethyl sulfoxide (DMSO) and then administered to the rats (3 mg/kg). At 4 weeks after 2VO surgery, all the groups were intraperitoneally injected with the corresponding solution once a day for 4 weeks. The MWM task was processed 50 days after 2VO. The overall experimental protocol is shown in Figure 1.

### Morris water maze task

The MWM task was conducted in all rats 22 days after drug administration to evaluate their spatial learning and memory retention. The MWM (ZS-001, ZSDichuang) consisted of a circular swimming tank with a diameter of 150 cm and depth of 60 cm. The tank was divided into four quadrants with different markers. A camera was installed above the tank to record the behavior of the rats. A 15 cm-wide circular platform was placed in the tank and submerged by opaque black water. Each testing rat was thrown into the water at one of the four quadrants and was given 120 s to find the platform. The rats that failed to locate the platform were guided to the platform and retained for 20 s. The rats were administered with the corresponding formulations after MWM test once a day during the protocol. The MWM task was conducted in two stages. The first stage was the navigation test. On the first day, the platform was visible to the rats and the time of reaching the platform was recorded. In the subsequent days, the rats were tested twice a day, and the platform was hidden. The tests were continuously repeated 4 days to examine the indicators of escape latency, swimming track, times of crossing the platform, time of staying at the aim quadrant and path length at the aim quadrant. The second stage of spatial probe test was performed on the sixth day, and the platform was removed. The values of each indicator were recorded and analyzed to assess the learning and memory performances of the rats. The evaluator conducting the MWM tests was blinded to the experiment protocol.

### Cerebral blood flow monitoring

CBF was monitored using a laser doppler perfusion and temperature monitor (Moor VMS-LDF1, Moor Instruments, UK). The rats of each group were anesthetized with chloral hydrate (3.5 mL/kg, i.p.). A paramedian skin incision was performed, and the subcutaneous tissue and cranial fascia were dissected to reach the skull bone. The probe of the monitor was placed at the settled skull bone area to detect the CBF (Cuccione et al., 2016). CBF changes in the rats were monitored before 2VO operation at the following successive periods: 1, 6, 24 h, 3, 7, 14, and 28 days after operation, as well as 1, 7, 14, and 28 days after drug treatment.

### Immunohistochemistry

The expression of the CB2 was determined by immunohistochemical staining in the brain as described in our previous study (Teng et al., 2016). The rats were anesthetized and then transcardially perfused with 0.9% sodium chloride and fixed with 4% paraformaldehyde. The brains were segregated and embedded in paraffin and subsequently sectioned. The sections were deparaffinized in xylene and rehydrated in ethanol solutions. The antigen was retrieved in boiled water bath using a 0.01 M citrate solution (pH 6.0) for 15 min. The sections were cooled down to room temperature and washed in PBS for 10 min before incubation in a goat serum blocking solution for 40 min. The sections were then incubated with primary antibodies directed against the CB2 [CB2 (H-60), diluted in 1:25, Santa Cruz, USA] overnight at 4°C. After washing with PBS, the sections were incubated with biotinylated goat anti-rabbit antibodies (1:200, Zhongshan Golden Bridge Biotechnology, ZSGB-Bio, China) for 30 min at 37°C The sections were counterstained with 3, 3-diaminobenzidine (DAB, ZSGB-Bio, China) and then incubated in PBS for use as negative controls. The immunohistochemically stained tissue sections were observed under a microscope. This procedure was performed by an author blinded to the experimental protocol. The number of positively immunostained cells in the CA1 region of the hippocampus was counted randomly in five microscopic fields at 400 × magnifications. The positive rate in the nerve fibers was eveluated using ImagePro plus software and represented as integrated optical density (IOD).

### Western blot analysis

Frozen hippocampus samples were mechanically homogenized in RIPA lysis buffer (Beyotime, China), and then centrifuged at 12,000 rpm for 15 min at 4°C. Protein concentration was determined using a BCA Protein Assay Kit (Beyotime). Proteins mixed with loading buffer were separated via 10% SDS-PAGE (Beyotime) and electrically transferred onto a polyvinylidene fluoride (PVDF) membrane (Millipore, USA; Lou et al., 2016). This membrane was then blocked with 5% bovine serum albumin (BSA) for 1 h at room temperature. The blocked membrane was incubated overnight at 4°C with primary antibodies directed against CB2 (Santa Cruz), phospho-Akt (p-Akt) (Cell Signaling, USA), Akt (Santa Cruz) and PI3K (Proteintech, China) diluted in Western blot primary antibody diluents at 1:200, 1:1000, 1:500, and 1:1000 respectively. After being washed thrice with PBS + Tween 20 (PBST), the membrane was incubated with horseradish peroxidase (HRP)-conjugated secondary antibody (diluted 1:3000 in PBST; Beyotime) for 1 h at 37°C. The immunoreactivity of the membrane was detected by chemiluminescence (ECL, Beyotime, China). Band densitometric analysis was performed using the ChemiDoc detection system and Quantity One software (Bio-Rad).

### TUNEL staining

A TUNEL staining was performed on the paraffin-embedded sections to assess neuronal apoptosis in the brain tissues. The sections were processed using an *in situ* cell death detection kit (Roche, Switzerland; Teng et al., 2016). The sections were dewaxed in xylene, ethyl alcohol, distilled water, and PBS and then incubated with Proteinase K, 0.1% sodium citrate and H_2_O_2_. After the slides were washed with PBS, they were incubated with 20 μL TUNEL reaction mixture at 37°C for 60 min. The sections were washed and incubated with 20 μL POD at 37°C for 60 min. TUNEL-positive cells were identified, counted, and analyzed under a light microscope (Leica, Germany) by a researcher who was blinded to the experimental groups. The level of brain damage was evaluated using the apoptotic index, which was the average number of positive cells counted in five microscopic fields at 400 × magnifications in each CA1 region of the hippocampus section.

### HE staining

After the MWM tests, rats were anesthetized and perfused through the left cardiac ventricle with PBS and then 4% paraformaldehyde. The brains were removed and fixed. The fixed tissues were embedded in paraffin. The brains were serially sliced to 5 μm-thick sections. The sections for hematoxylin-eosin (HE) staining were placed onto the uncoated slides. The sections were then HE stained routinely for histomorphological assessment (Xu et al., 2012). The evaluator assessing the histology was blinded to the experiment protocol.

### Statistical analysis

All experimental results were presented as mean ± standard deviation (*SD*). All the experimental data were statistically analyzed by SPSS 17.0. The group differences in the tests were analyzed by one-way analysis of variance (ANOVA) or Student's *t*-test. The *P* < 0.05 was considered statistically significant.

## Results

### Preparation and evaluation of HPβCD/BCP

#### Preparation and optimization of HPβCD/BCP

The inclusion efficiency of different prescriptions ranged from 12.3 to 56.9% and was determined from the 20 experimental runs generated by the central composite design. The effects of the three independent variables (X_1_, X_2_, and X_3_) on the inclusion complex were examined by analyzing the inclusion efficiency values.

**Figure 2:**
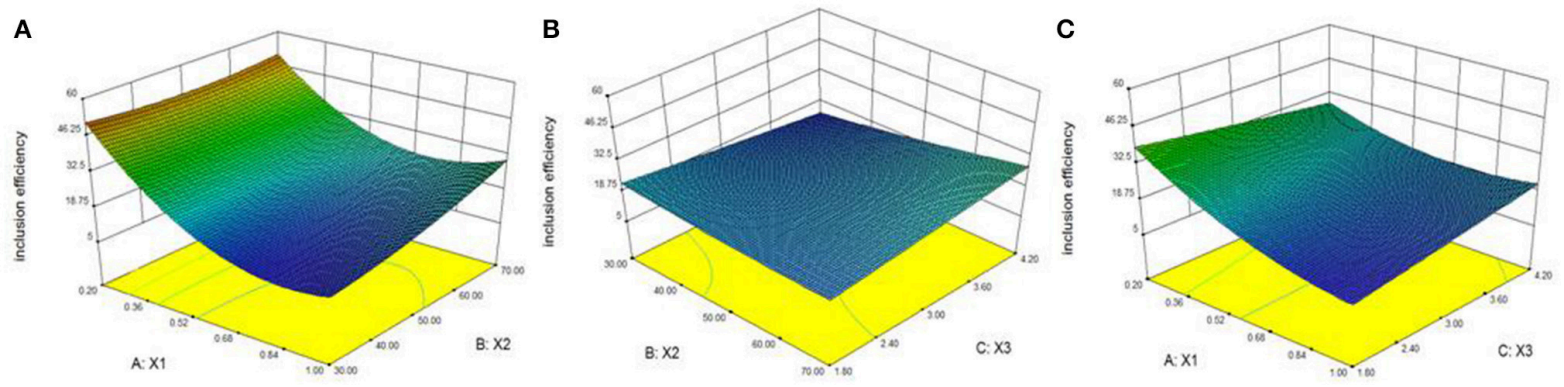
**Response surface plots showing effects of material inputting ratio (X_**1**_), reaction temperature (X_**2**_), and reaction time (X_**3**_) on inclusion efficiency**. The inclusion efficiency of inclusion complex increased when X1 and X3 decreased while X2 increased. **(A)** Effects of X1 and X2 on inclusion efficiency. **(B)** Effects of X2 and X3 on inclusion efficiency. **(C)** Effects of X1 and X3 on inclusion efficiency.

Figure 2 shows the effects of X1, X2, and X3 on the inclusion efficiency. The results indicated that the inclusion efficiency increased when X1 and X3 decreased while X2 increased. Considering the stability of BCP, we determined that the optimized formulation achieved a material inputting ratio of BCP and HPβCD at 0.2, reaction temperature of 50°C and reaction time of 3 h. The optimized inclusion complex showed an inclusion efficiency of 56.9%.

#### Validation of HPβCD/BCP

**Figure 3:**
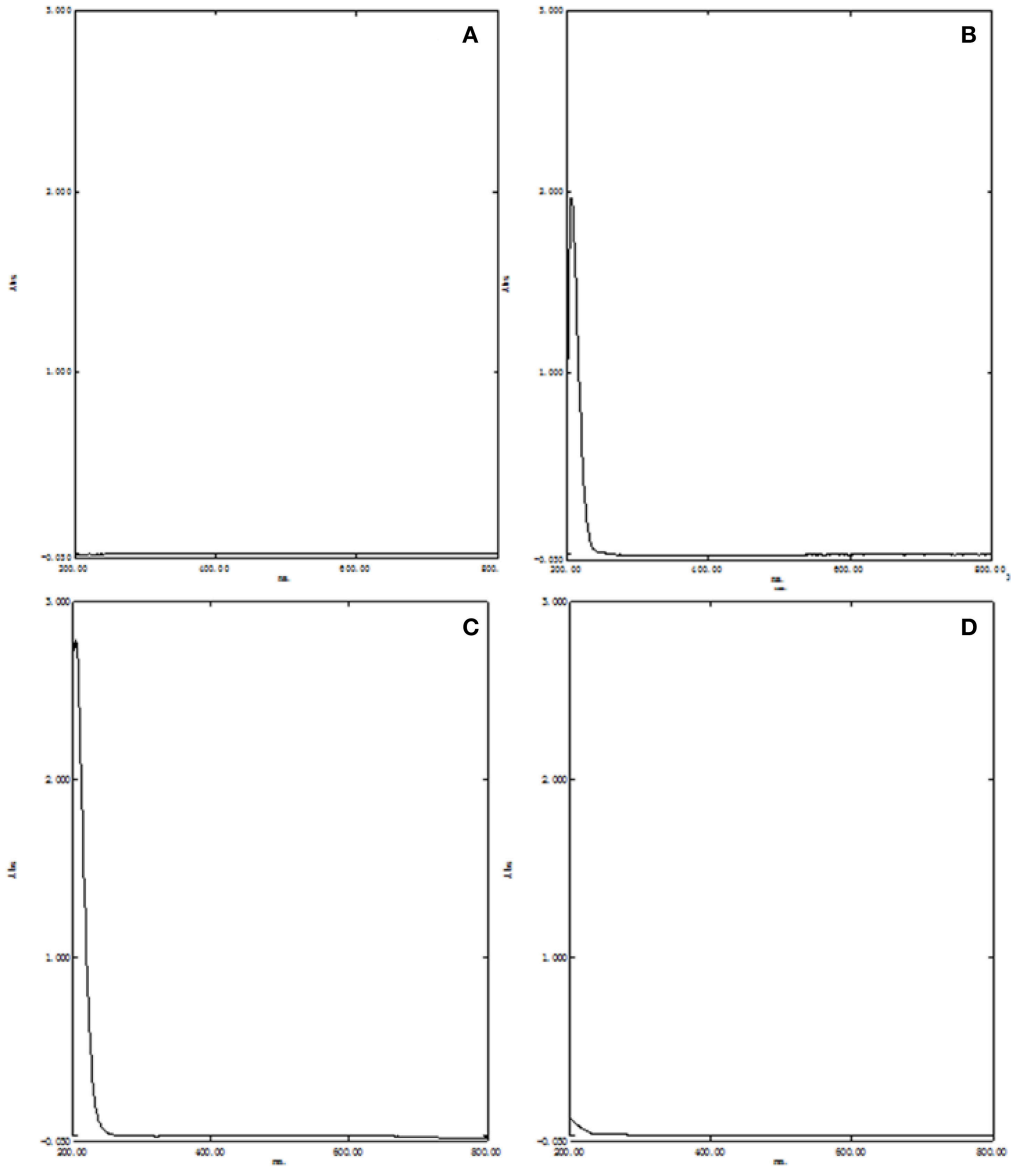
**UV–vis absorption spectra of (A)** HPβCD, **(B)** BCP, **(C)** physical mixture, and **(D)** HPβCD/BCP. No absorption peak is observed in HPβCD. The spectra of BCP and the physical mixture were identical, and both achieved a maximum absorption wavelength of 205 nm. No absorption peak was found in the spectrum of the HPβCD/BCP.

UV–vis spectrophotometry, DSC, and FT-IR were used to validate the formation of HPβCD/BCP. UV–vis absorption spectra were acquired and recorded for HPβCD, BCP, the physical mixture, and the HPβCD/BCP. No absorption peak is observed in Figure 3A. The UV–vis spectra of BCP (Figure 3B) and the physical mixture (Figure 3C) were identical, and both achieved a maximum absorption wavelength of 205 nm. However, no absorption peak was found in the spectrum of the HPβCD/BCP (Figure 3D). The results indicated that BCP had been wrapped into the HPβCD, forming an inclusion complex with the latter.

**Figure 4:**
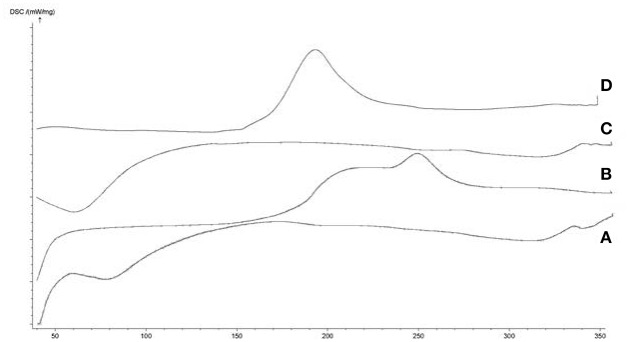
**DSC curves of (A)** BCP, **(B)** HPβCD, **(C)** physical mixture, and **(D)** HPβCD/BCP. BCP generated a wide exothermic peak from 180 to 260°C. HPβCD exhibited an endothermic peak at 60°C. An exothermic peak of physical mixture appeared at 190°C. An endothermic peak of HPβCD/BCP appeared at 70°C, but no peak showed up from 180 to 260°C.

DSC is an important technique that can verify the formation of inclusion complex. The melting, boiling, and sublimation points of the drug molecules either shift to different temperatures or disappear when these molecules are inserted in the HPβCD cavities. The thermograms of HPβCD, BCP, the physical mixture, and the HPβCD/BCP are shown in Figure 4. The absence of the peak of BCP in the thermogram of HPβCD/BCP may be attributed to the inclusion of BCP into the HPβCD cavity, suggesting the formation of the HPβCD/BCP.

**Figure 5:**
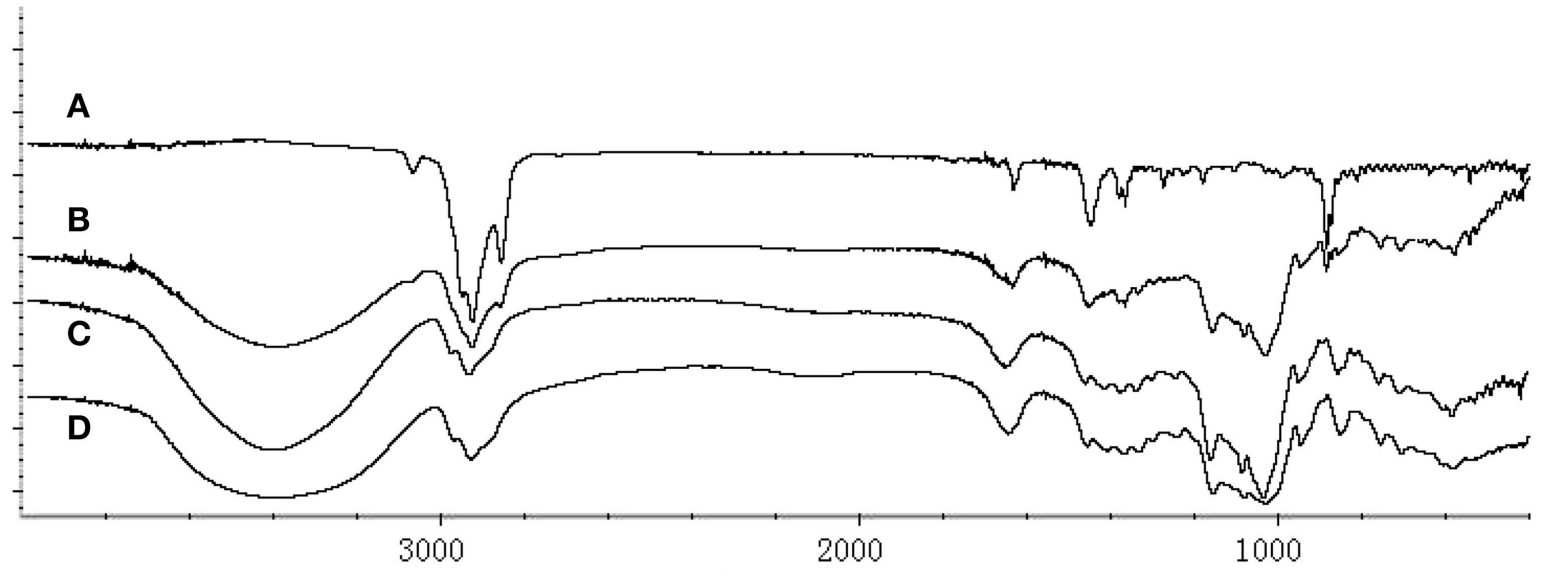
**FT-IR spectra of (A)** BCP, **(B)** physical mixture, **(C)** HPβCD, and **(D)** HPβCD/BCP. The BCP spectrum showed the bands from 3070 to 2680 cm^−1^ and 1637 to 870 cm^−1^. The physical mixture presented the absorption band of BCP at 885 cm^−1^. HPβCD showed a absorption bands at 3401 cm^−1^. HPβCD/BCP showed only a few identical features compared with BCP.

The HPβCD/BCP was characterized by FT-IR spectroscopy. The spectra of HPβCD, BCP, HPβCD/BCP, and their physical mixture from 4000 to 400 cm^−1^ are presented in Figure 5. The BCP spectrum showed the bands at 3070–2680 and 1637–870 cm^−1^ (Figure 5A). The spectrum of the physical mixture (Figure 5B) presented a weak characteristic absorption band of BCP at 885 cm^−1^. The spectrum of HPβCD (Figure 5C) showed a remarkable absorption bands at 3401 cm^−1^ (Tang et al., 2015). However, compared with the spectrum of the HPβCD/BCP few features were observed to be identical to BCP (Figure 5D). The main differences can be ascribed to the formation of the inclusion complex related to the intramolecular hydrogen bonds between BCP and HPβCD molecules.

#### Evaluation of HPβCD/BCP

**Figure 6:**
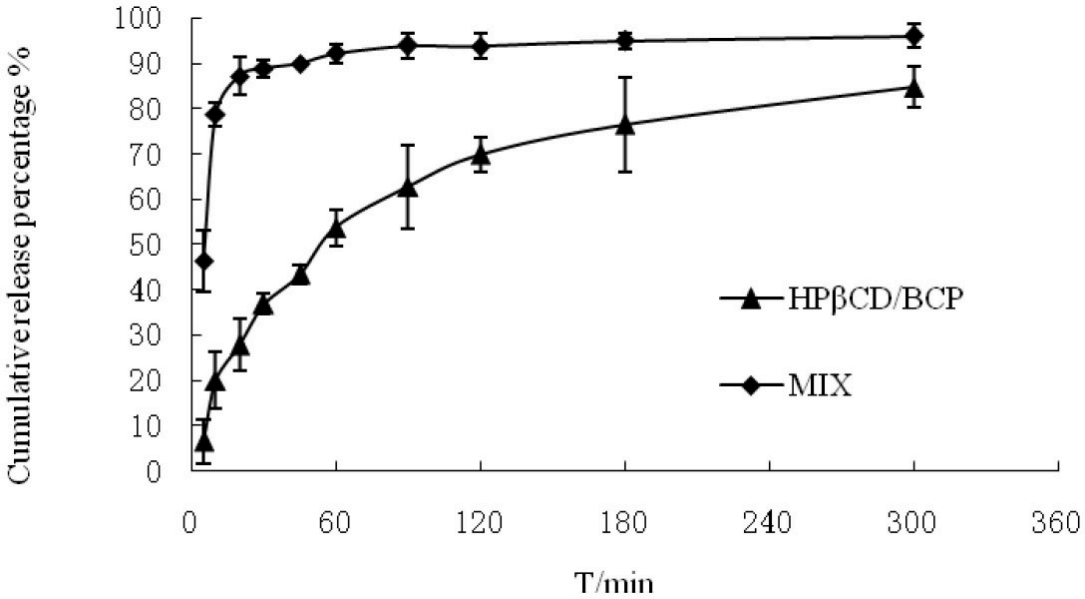
**Mean dissolution profiles of HPβCD/BCP and their physical mixture in the mediums phosphate buffer (pH 6.8) (***n*** = 3)**.

The HPβCD/BCP was evaluated both *in vitro* and *in vivo*. Figure 6 shows the dissolution profiles of the HPβCD/BCP and their physical mixture in PBS (pH 6.8). They were characterized by an initial fast release followed by a relatively slow release until a constant value.

**Figure 7:**
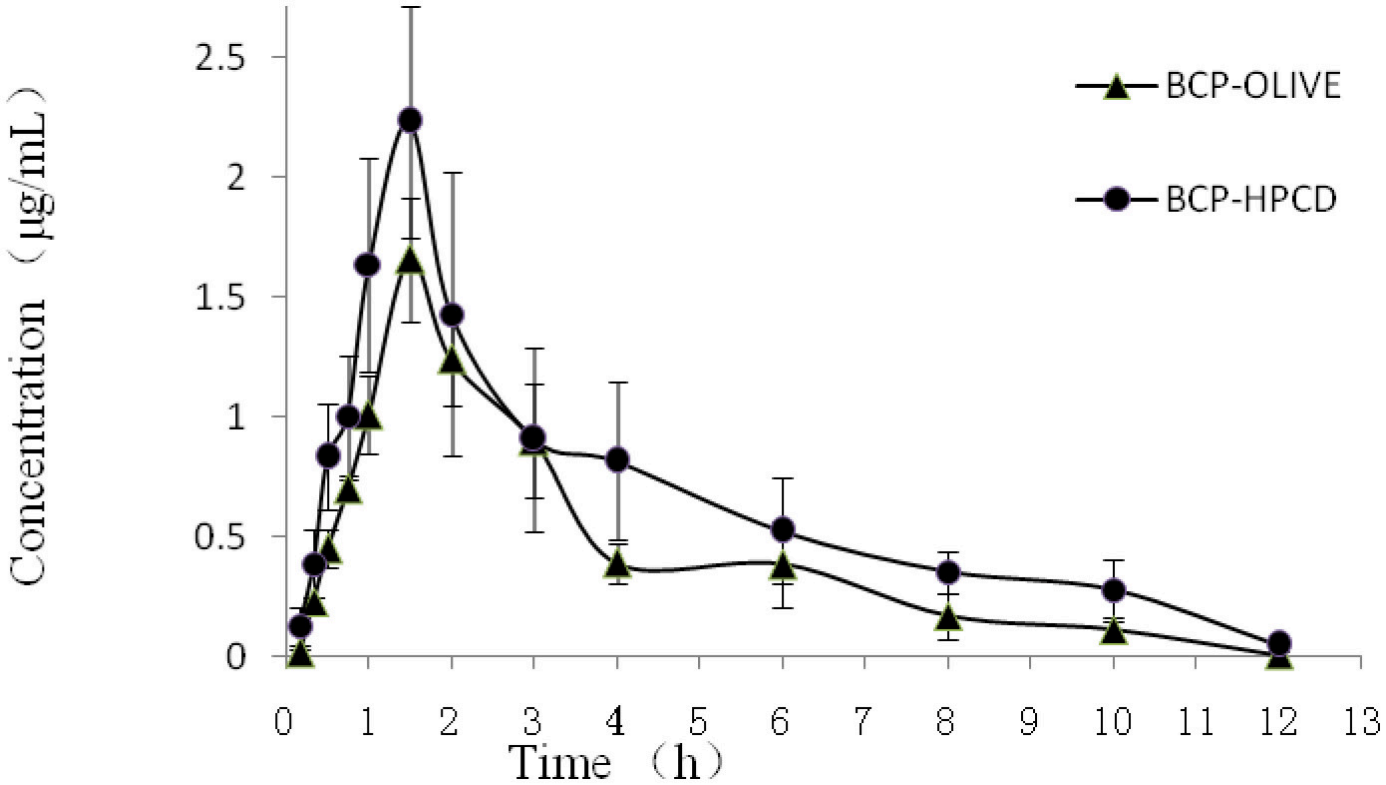
**Mean (±S.D.) plasma concentration–time profiles of BCP-OLIVE and HPβCD/BCP by intraperitoneal injection at a dose of 50 mg/kg (***n*** = 5)**. The plasma content of BCP administered with HPβCD/BCP was higher at all time points in comparison to that obtained after administered with BCP-OLIVE.

The results of pharmacokinetic study of BCP-OLIVE and HPβCD/BCP are shown in Figure 7. The plasma content of BCP administered with HPβCD/BCP was significantly higher at all time points in comparison to that obtained after injection of BCP-OLIVE. The relevant pharmacokinetic parameters of BCP in rat plasma after injection with the two formulations were calculated. The parameters are summarized in Table 2. The results demonstrated that HPβCD/BCP prominently increases the bioavailability of BCP in rats as compared with BCP-OLIVE.

**Table 2:** **Pharmacokinetic parameters of different formulations**.

**Pharmacokinetic parameters**	**BCP-OLIVE**	**HPβCD/BCP**
Cmax (μg/ml)	1.7578 ± 0.2069	2.7796 ± 0.3445
Tmax (h)	1.7000 ± 0.1225	1.6000 ± 0.1871
T_1/2_(h)	2.0046 ± 0.4276	2.6168 ± 0.3858
MRT (h)	3.0996 ± 0.2942	3.8094 ± 0.2696
AUC _0−12*h*_ (μg^*^h/ml)	5.0086 ± 0.8860	9.0264 ± 1.6495
AUC0-∞(μg^*^h/ml)	5.4722 ± 0.0815	9.6428 ± 1.5587

### Protective effects of HPβCD/BCP in 2VO model

**Figure 8:**
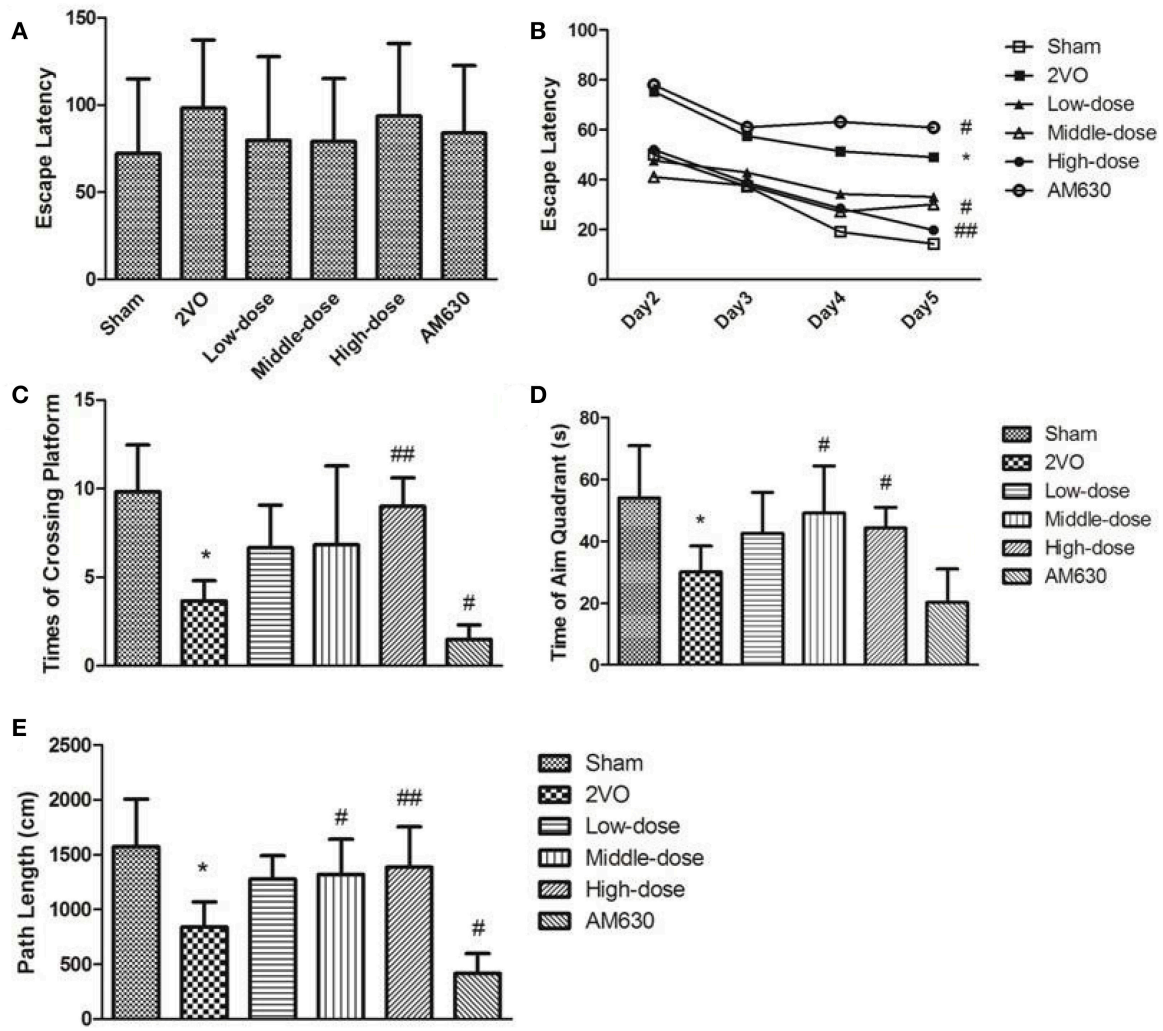
**MWM test results of rats' performance. (A)** Visible platform task on the first day. **(B)** Mean escape latency calculated for each day in sham, 2VO, BCP treated low-dose, middle dose, high dose and AM630 treated group in navigation test. Results of **(C)** times of crossing platform, **(D)** time of aim quadrant, **(E)** path length of each group in spatial probe tests. All the rats reached the platform in the visible platform task on the first day. The rats in the 2VO group and AM630 group performed longer escape latencies than other groups in the MWM training. Compared with rats in the sham group and BCP treated groups, rats in the 2VO group and AM630 group exhibited less times of crossing platform, time of staying at the aim quadrant and path length at aim quadrant. (^*^P < 0.05 vs. sham group, ^#^*P* < 0.05 vs. 2VO group, ^##^*P* < 0.001 vs. 2VO group).

The effects of BCP on the spatial learning and memory of rats were examined through the MWM test. All the rats without training reached the platform in the visible platform task on the first day (Figure 8A). In the MWM training, the rats in the 2VO group and AM630 group performed longer escape latencies than the rats in the sham group and HPβCD/BCP treated group (Figure 8B). The abilities of spatial learning and memory retention of rats were evaluated using the times of crossing platform, time of staying at the aim quadrant and path length at aim quadrant in the spatial probe tests performed on the sixth day. Compared with rats in the sham group, rats in the 2VO group exhibited less times of crossing platform, time of staying at the aim quadrant and path length at aim quadrant (*P* < 0.05 all). The spatial learning and memory of rats in the HPβCD/BCP treated group improved significantly compared with rats in the 2VO group, especially in the middle and high dose groups (*P* < 0.05 in middle-dose and *P* < 0.001 in high-dose vs. 2VO group). The times of crossing the platform and path length at aim quadrant of rats in the AM630 group were apparently lower than those of the 2VO group (*P* < 0.05, Figures 8C,E). Although the test of time of staying at aim quadrant showed no significant difference between the two groups (*P* > 0.05), a decreasing trend was observed in the AM630 group (Figure 8D).

**Figure 9:**
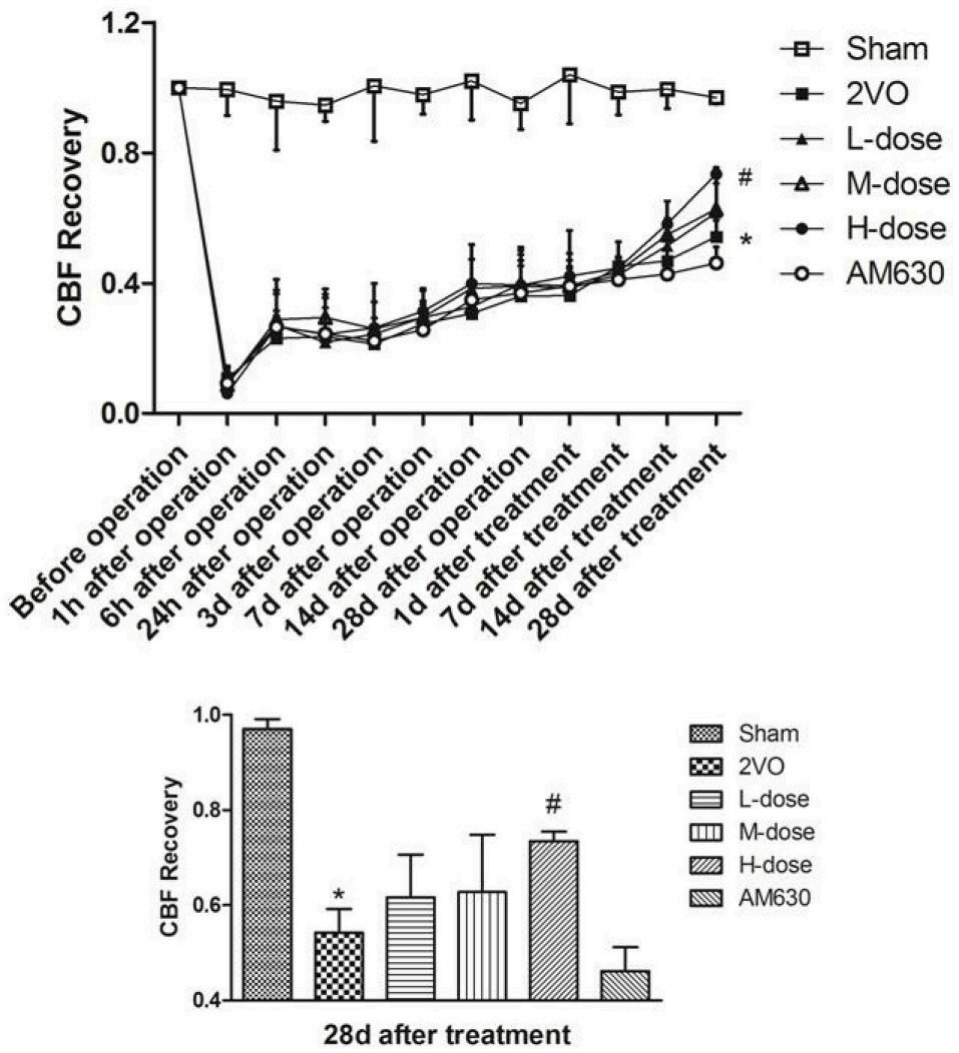
**CBF monitoring results of each group**. The CBF recoveries of the different groups were approximate before drug treatment, while the recoveries rised rapidly after the drug treatment. At the 28th day after drug treatment, the CBFs of rats in the high dose HPβCD/BCP treated group recovered faster than those in the 2VO group, and the AM630 group presented the slowest recovery. (^*^P < 0.001 vs. sham group, ^#^*P* < 0.05 vs. 2VO group).

The CBF values of the rats before 2VO operation, after operation and after drug treatment were monitored to detect the effects of HPβCD/BCP on CBF (Figure 9). All tests results were expressed as CBF recoveries, which were calculated as the percentage of the residual perfusion after 2VO compared with pre-operation baseline. CBF of all rats declined distinctly after 2VO except that in the sham group. After the operation, the CBF recovered relatively slowly. The recoveries of the different groups were approximate before drug treatment and slowly rebounded to about 36.1–39.7% at 28 days after operation. After the drug treatment, the recoveries continued to rise rapidly. At the 28th day after drug treatment, the CBF of rats in 2VO was still lower than that of the sham-operated rats (*P* < 0.001), but the CBFs of rats in the high dose HPβCD/BCP treated group recovered faster than those in the 2VO group (*P* < 0.05). The CBF recovery in low- and middle-dose HPβCD/BCP treated group did not show significant statistical differences with those in the 2VO group (*P* > 0.05). However, a faster recovery rate was observed in the HPβCD/BCP treated groups. The CBF in the AM630 group presented the slowest recovery among all the groups.

**Figure 10:**
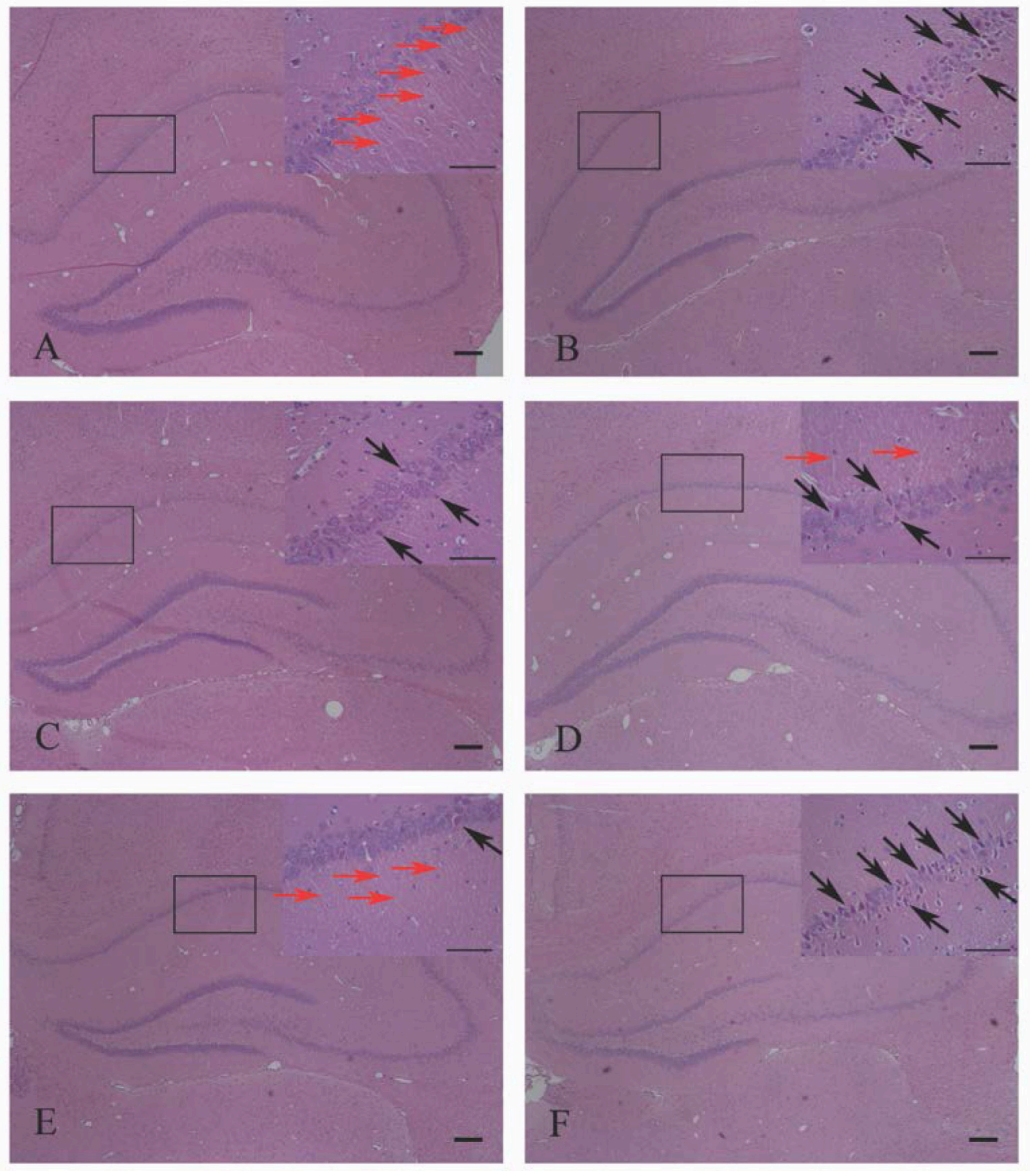
**HE staining showed the effects of HPβCD/BCP on morphologic changes in hippocampal neurons caused by 2VO: sham group (A)**, 2VO group **(B)**, HPβCD/BCP low dose group **(C)**, HPβCD/BCP middle dose group **(D)**, HPβCD/BCP high dose group **(E)**, and AM630 group **(F)**. In the 2VO group, neurons were paramorphia, their nuclei became triquetrous or polygonous, and the nerve fibers were fractured and reduced. In the HPβCD/BCP group, less abnormal neurons were observed. In the AM630 group, the neurons were triquetrous and wedge-shaped, the nuclei were irregular, the cytoplasm was aggregated and the nerve fibers were fractured and reduced. The red arrows showed the nerve fibers and the black arrows showed the abnormal neurons. The bar is 100 μm.

HE staining was used to detect the effects of HPβCD/BCP on the morphological changes in the hippocampal neurons of the CA1 region. In the sham group, morphology of the hippocampal neurons was inerratic, the nuclei were mellow and full, stained in blue, endochylema was completely filled and stained in pink (Figure 10A). Figure 10B shows the hippocampus in the rats in the 2VO group. In these hippocampus, some neurons exhibited paramorphia and were stained in blue, and their nuclei became triquetrous or polygonous with irregular morphology. The cytoplasms of the variant neurons were stained in dark red or dark violet, and the nerve fibers were fractured and reduced. In the hippocampus of the HPβCD/BCP treated rats, the number of abnormal neurons was less than that in the 2VO rats. In addition, the nuclei and cytoplasm of their neurons were polygonous, their colors, and morphologies slightly changed (Figures 10C–E). In the hippocampus of the AM630 treated rats (Figure 10F), the neurons were distinctly abnormal, and most of them were triquetrous and wedge-shaped, some neurons were fusiformis in shape. The nuclei were highly irregular and darkly stained. The cytoplasm was aggregated and stained in dark violet. The nerve fibers in the AM630 group were obviously fractured and reduced.

**Figure 11:**
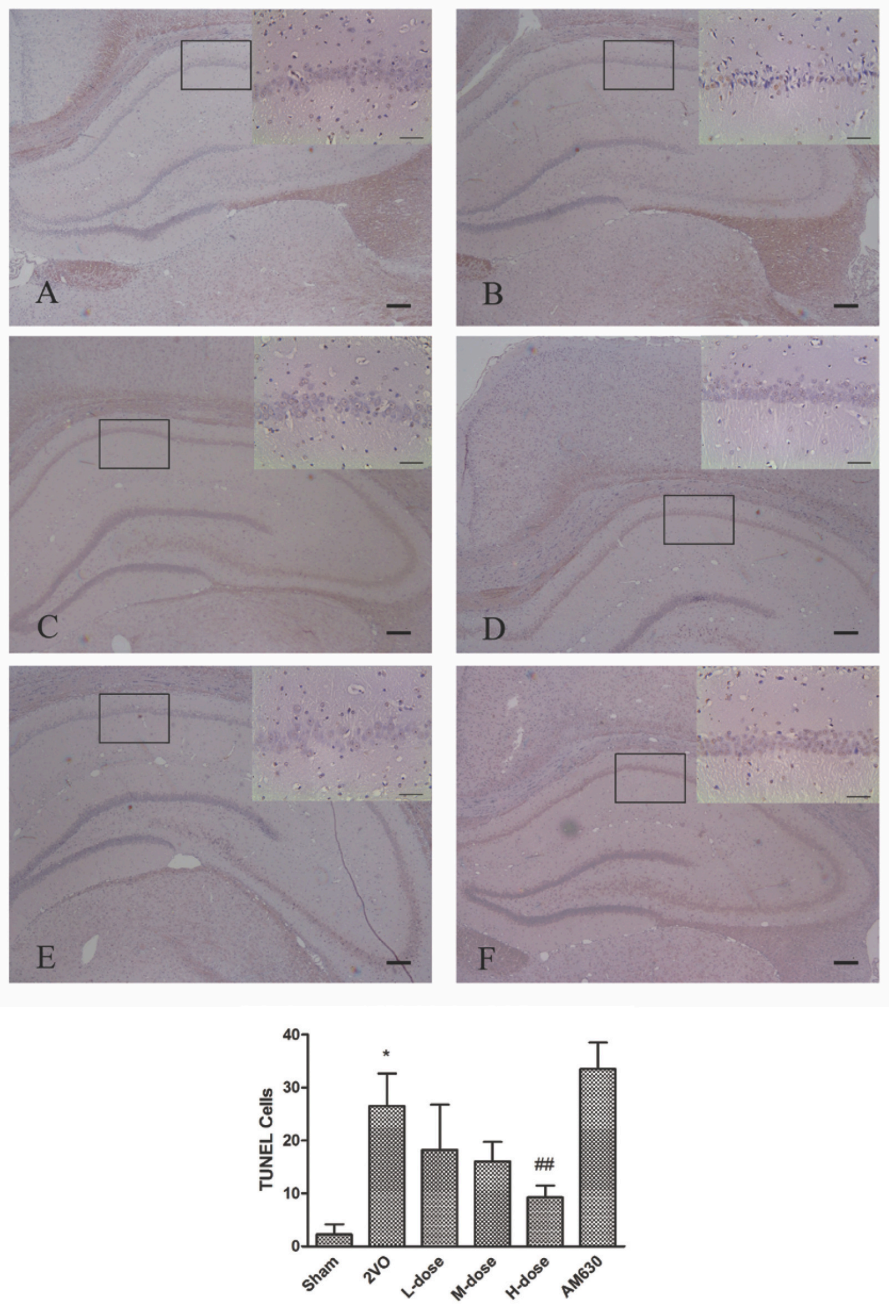
**TUNEL staining showed the effects of HPβCD/BCP on apoptosis in hippocampal neurons caused by 2VO: sham group (A)**, 2VO group **(B)**, HPβCD/BCP low dose group **(C)**, HPβCD/BCP middle dose group **(D)**, HPβCD/BCP high dose group **(E)**, and AM630 group **(F)**. The TUNEL positive cells in the 2VO group significantly increased in the CA1 region of hippocampus compared with the sham group. After HPβCD/BCP treatment, the number of TUNEL positive cells was obviously suppressed. And AM630 aggravated apoptosis in the hippocampus compared with the 2VO rats. The bar is 100 μm (^*^P < 0.001 vs. sham group, ^##^*P* < 0.001 vs. 2VO group).

The effects of HPβCD/BCP on the apoptosis of the hippocampal neurons caused by 2VO are presented in Figure 11. The TUNEL positive cells were darkly stained and regarded as apoptotic cells. Compared with the sham group, the TUNEL positive cells in the 2VO group significantly increased in the CA1 region of hippocampus (*P* < 0.01, Figure 11B). After HPβCD/BCP treatment, the number of darkly stained cells was obviously suppressed relative to that in the 2VO group, which was exerted in a dose-dependent manner (*P* < 0.05 in low- and middle-dose groups, and *P* < 0.001 in high-dose group, Figures 11C–E). The CB2 inhibitor, AM630, can further aggravate apoptosis in the hippocampus compared with the 2VO rats, although no statistical difference was observed (*P* = 0.71, Figure 11F).

### Effect of HPβCD/BCP on the CB2 pathway after 2VO

**Figure 12:**
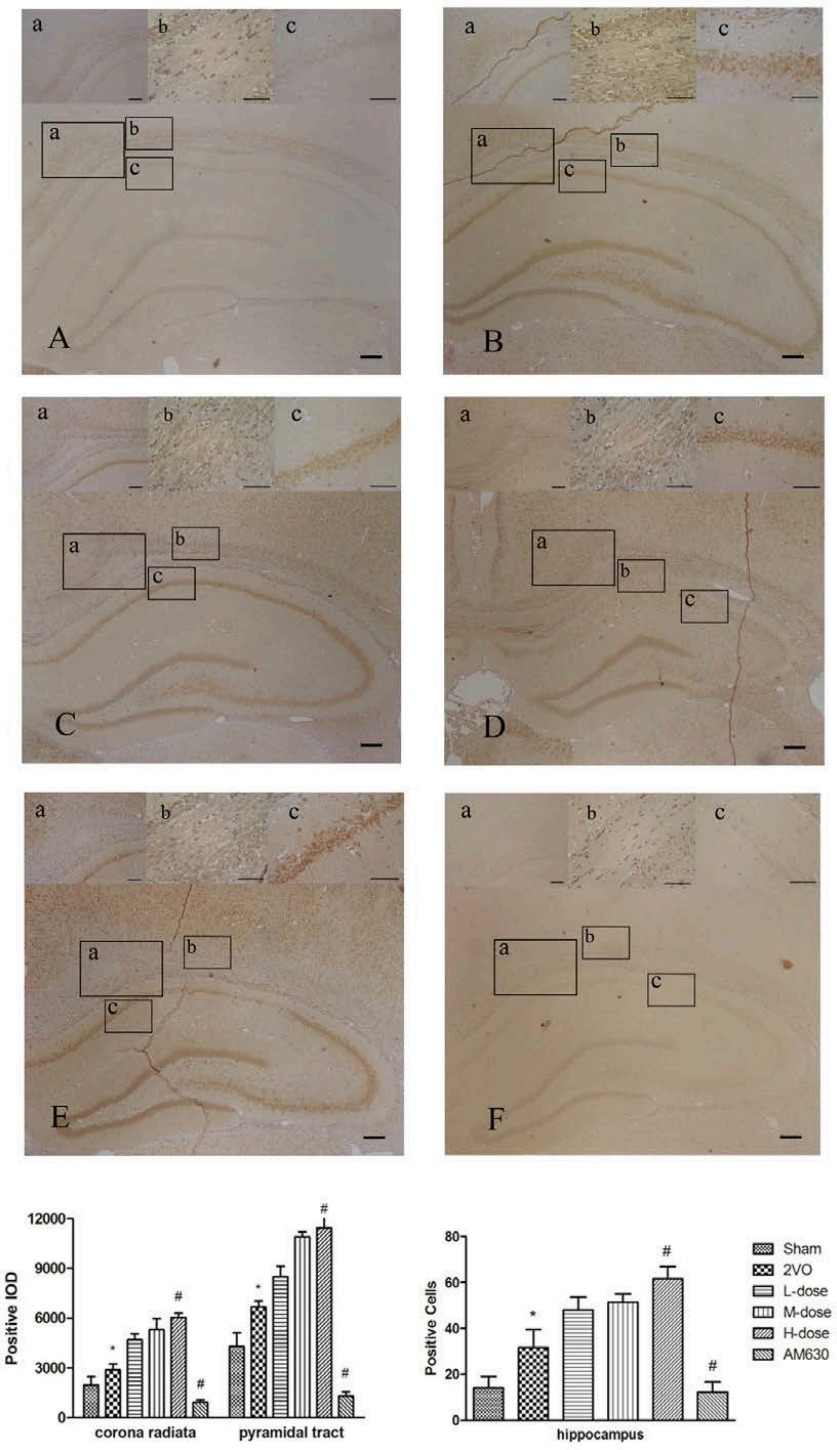
**Immunohistochemical staining showed the expressions of CB2 in corona radiata (a), pyramidal tract (b) and hippocampus CA1 region (c) in different groups: sham group (A)**, 2VO group **(B)**, BCP low dose group **(C)**, BCP middle dose group **(D)**, BCP high dose group **(E)**, and AM630 group **(F)**. The expression levels of the CB2 increased in the 2VO group compared with those in sham group. The HPβCD/BCP significantly increased the expression levels of CB2, and the CB2 expression levels increased with the increase of BCP concentration. In the AM630 group, the CB2 presented extremely low expression levels. The bar is 100 μm (^*^P < 0.01 vs. sham group, ^#^*P* < 0.001 vs. 2VO group).

The expression levels of the CB2 were detected by immunohistochemical staining (Figure 12). In the brain tissues of sham-operated rats, the CB2 were mainly expressed in the cytoplasm of the neurons and nerve fibers (Figure 12A). In the 2VO group (Figure 12B), the expression levels of the CB2 significantly increased in the corona radiata (a), pyramidal tract (b) and hippocampus (c). The HPβCD/BCP group significantly increased the CB2 after 2VO operation compared with the 2VO group, and the CB2 expression levels increased with the increase of BCP concentration (Figures 12C–E). However, in the AM630 group, the CB2 presented extremely low expression levels in the brain tissue, which verified the suppression effect of AM630 (Figure 12F).

**Figure 13:**
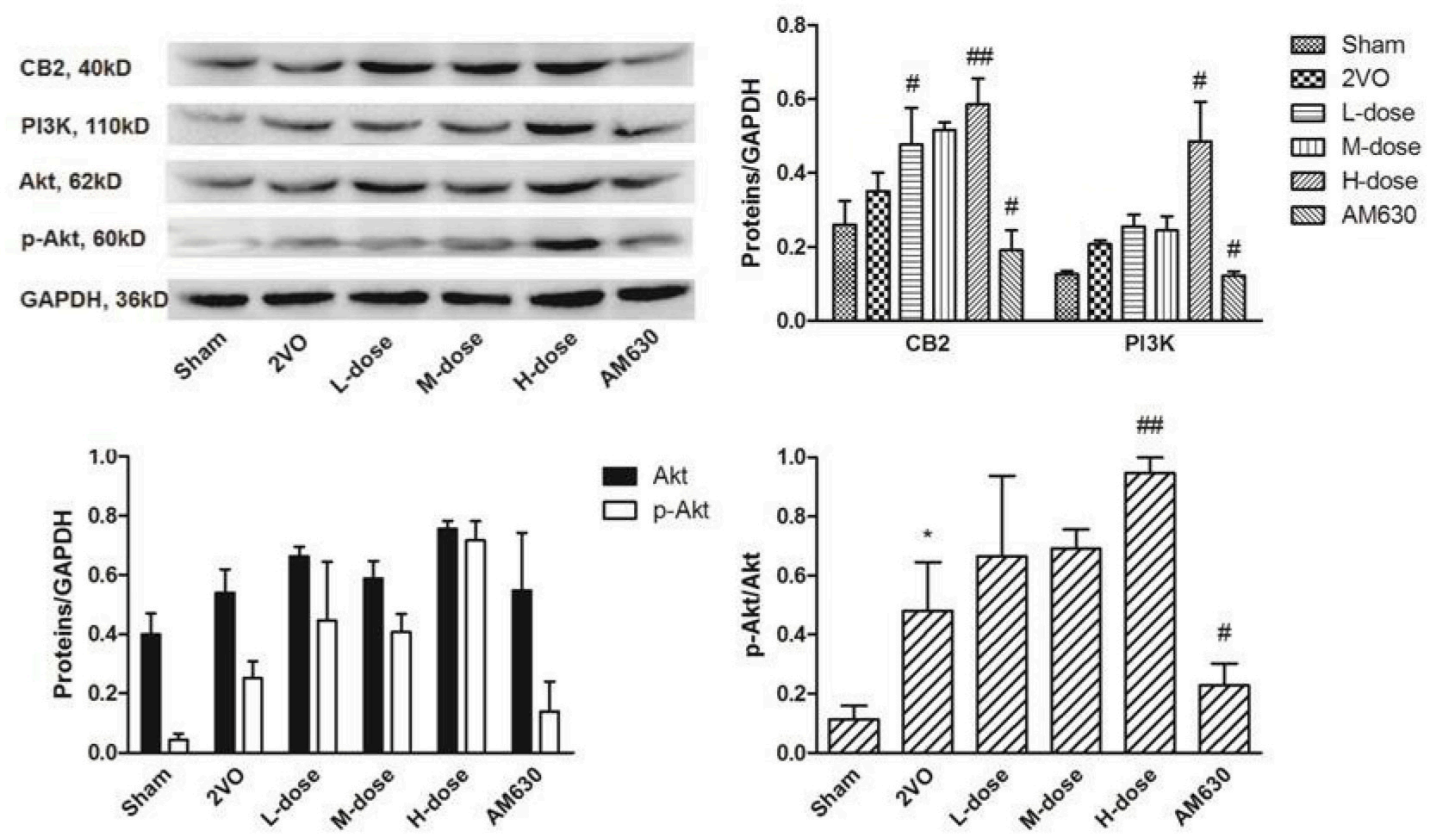
**The expressions of CB2, PI3K and Akt in rats' brain tissues of different groups**. The expression levels of the CB2 and PI3K increased slightly in the 2VO group compared with those in the sham group. After HPβCD/BCP treatment, the expression levels of CB2 and P13K, p-Akt increased compared with those of the 2VO group, whereas these levels decreased in the AM630 treated group. (^*^P < 0.05 vs. sham group, ^#^*P* < 0.05 vs. 2VO group, ^##^*P* < 0.01 vs. 2VO group).

The expression levels of the CB2, PI3K, Akt, and p-Akt were detected by Western blot (Figure 13). Although the expression levels of the CB2, as well as PI3K, increased slightly in the 2VO group compared with those in the sham group, the expression levels in these groups showed no statistical difference. After HPβCD/BCP treatment, the CB2 expressed incrementally with the incremental dose of BCP (*P* < 0.05 in low- and middle-dose groups vs. 2VO group, *P* < 0.01 in high-dose group vs. 2VO group). AM630 inhibited the expression of the CB2 as indicated by the reduced CB2 expression in the 2VO group (*P* < 0.05), and this finding corresponded to the immunohistochemical staining result. The expression levels of PI3K and p-Akt were similar to that of CB2. Compared with those of the 2VO group, the expression levels of the P13K, p-Akt, and CB2 increased in the HPβCD/BCP treated groups, especially in the high-dose group (*P* < 0.05 in PI3K, and *P* < 0.01 in p-Akt), whereas these levels decreased in the AM630 treated group (*P* < 0.05). These results indicated that the activation of the CB2 regulated the expression levels of PI3K and p-Akt.

## Discussion

In this study, we successfully developed the HPβCD/BCP, which will be used for subsequent research. The inclusion complex was prepared by solution-stirring and freeze-drying method and then evaluated by UV–vis spectrophotometry, DSC, FT-IR, *in vitro* dissolution study and *in vivo* pharmacokinetic study. The MWM test and CBF monitoring confirmed the establishment of VD model by 2VO. In addition, the HPβCD/BCP can improve the learning and memory abilities in rats and promote the recovery of CBF. Molecular biology experiments proved that HPβCD/BCP can alleviate the injury of neurons and nerve fibers, and its effect may be promoted by upregulating the CB2-PI3K-Akt pathway.

VD induced by chronic cerebral ischemia is often characterized by a progressive cognitive deterioration induced by blood reduction in vulnerable brain regions. Previous studies found that a decrease in CBF can damage the neurons in the vulnerable regions of the brain, especially in the hippocampus and white matter (Kitamura et al., 2016). Damage in the hippocampus neurons and loss of white matter fibers can disrupt the learning ability and cause memory, which in turn can result in dementia. Damage to the hippocampus can cause memory loss (Clark et al., 2007). Given that spatial learning and memory were dependent on the integrity of the hippocampus (Xu et al., 2012), deficits in spatial learning and memory were found in the presence of lesions in the hippocampus (Clark et al., 2007). Moreover, cerebral white matter is extremely vulnerable to ischemia. Damage to white matter is mainly manifested as injury to oligodendrocytes and distruction of myelinated axons (Kitamura et al., 2012). Lesions in white matter can disrupt nerve conduction, and this effect may result in dementia. In our study, the rats that underwent 2VO operation exhibited declined learning and memory abilities in the MWM test, as well as low CBF level. HE and TUNEL staining indicated that VD rats exhibited increased neurons paramorphia, apoptosis, degeneration, and loss of nerve fibers.

BCP generates neuroprotective effects against ischemic diseases and AD models (Choi et al., 2013; Cheng et al., 2014) through its anti-inflammation, anti-apoptosis, antioxidation effects, as well as the activation of CB2. However, studies on the effects of BCP on VD are few. Despite the effectiveness of BCP in neuroprotection, its properties such as poor water-solubility, volatility, and sensitivity, decrease the bioavailability and limit the pharmacologic action of the drugs. In our previous study, incommodity was observed during drug administration. BCP was usually dissolved in polyoxyethylated castor oil or olive oil, hence, inaccurate dosages and several inconveniences in the experimental operation are often encountered (Lou et al., 2016). In the present study, the HPβCD/BCP was confirmed to be effective and convenient. Thus, HPβCD/BCP was administrated in rats with cognitive deficits for 4 weeks. The MWM test and CBF monitoring results indicated that the HPβCD/BCP treatment improved the learning and memory of the rats and accelerated the recovery of their CBFs. These results suggested that HPβCD/BCP elicits a cerebral protective effect in a dose-dependent manner. Furthermore, no lesion was observed in the brains of the rats in the sham group, indicating that HPβCD exerted no effect on the BCP functions. Therefore, BCP played a neuroprotective role by improving the blood supply in the brain.

BCP was reported to be a CB2 selective agonist (Gertsch et al., 2008; Al Mansouri et al., 2014; Bahi et al., 2014), thus, it might elicit its neuroprotective effects by activating the CB2. The CB2, which was used to be known as the peripheral cannabinoid receptor type, was initially found in the immune system, but was recently detected in the central nervous system (Morgan et al., 2009; Lozovaya et al., 2011; Onaivi, 2011). In the present study, the immunohistochemistry results showed that CB2 were expressed in brain tissues, especially in neurons and nerve fibers. Moreover, the expression of CB2 can be increased by BCP in a dose-dependent manner. Administration of CB2 agonist can reduce the infract size of the brain and motor functional deficits and restrain the production of adhesion factor and matrix metalloproteinases, inhibit the inflammatory response, and improve the microcirculation after cerebral ischemia (Fernández-Ruiz et al., 2007; Benito et al., 2008; Pini et al., 2012). The CB2 can be upregulated in multiple central nervous system diseases, such as Alzheimer's disease, Parkinson's disease, Huntington's disease, and stroke (Capettini et al., 2012; Javed et al., 2016). The CB2 signaling mechanism is associated with the activation of the phosphatidylinositol 3-kinase (PI3K–Akt) pathway (Molina-Holgado et al., 2002; Palazuelos et al., 2006; Fernández-Ruiz et al., 2007). The PI3K/Akt pathway exhibits neuroprotection against brain disorders (Enriquez-Barreto et al., 2014; Gross and Bassell, 2014) and is associated with learning and memory abilities.

In the present study, the CB2 was observed to be mainly expressed in the cytoplasms of neurons and nerve fibers. After 2VO operation, expression of CB2 slightly increased possibly because of the compensatory protection mechanism. BCP treatment can upregulate the expression of CB2 by activating the PI3K/Akt pathway. However, AM630, a selective CB2 antagonist, downregulated the expression of the CB2 and suppressed the PI3K/Akt pathway, thus aggravating the ischemic injury.

In conclusion, we prepared a novel drug delivery system for BCP-HPβCD/BCP to improve its bioavailability. Administration of the HPβCD/BCP alleviated the cognitive dysfunction, improved the cerebral blood supply, suppressed neuronal apoptosis, and reduced the loss of nerve fibers in VD model rats. BCP possibly elicits its neuroprotective effects by activating the CB2 pathway. Therefore, the present results may be of considerable significance for targeted therapy and provide an innovative treatment strategy for vascular dementia. Further studies are required to determine the safety and possible side effects of HPβCD/BCP and explore the detailed mechanism of BCP on cognitive impairment.

## Ethics statement

This study was carried out in accordance with the recommendations of the Animal Experimental Committee of Chongqing Medical University with written informed consent from all subjects. All subjects gave written informed consent in accordance with the Declaration of Helsinki. The protocol was approved by the Animal Experimental Committee of Chongqing Medical University.

## Author contributions

LX and ZD designed experiments; JL, ZT, XT, RA, MY, and QZ carried out experiments; LZ and LM analyzed experimental results. JY and FW carried out gas chromatographic analysis. JL and ZT wrote the manuscript.

### Conflict of interest statement

The authors declare that the research was conducted in the absence of any commercial or financial relationships that could be construed as a potential conflict of interest.
